# The Fentanyl System Shock – Are There Lessons to Learn From the COVID-19 System Shock Framework? Comment on "The COVID-19 System Shock Framework: Capturing Health System Innovation During the COVID-19 Pandemic"

**DOI:** 10.34172/ijhpm.2023.7409

**Published:** 2023-03-05

**Authors:** Maximilian Meyer, Jean N. Westenberg, R. Michael Krausz

**Affiliations:** ^1^University Psychiatric Clinics Basel, University of Basel, Basel, Switzerland; ^2^Department of Psychiatry, University of British Columbia, Vancouver, BC, Canada

**Keywords:** Opioid Overdose Crisis, Fentanyl, COVID-19, Canada

## Abstract

The Sydney Children’s Hospitals Network (SCHN) addressed the challenges of the COVID-19 pandemic by implementing innovative changes which made their health system resilient and responsive. For other healthcare systems, there are important takeaways. In the United States and Canada, an urgent widespread response is needed to address the overdose crisis, driven by potent synthetic opioids (ie, fentanyl and its derivates). We project the COVID-19 System Shock Framework (CSSF) on to the North American healthcare systems and suggest a Fentanyl System Shock Framework, which provides a framework for necessary changes and innovations to address the overdose crisis. To become resilient to the fentanyl system shock, core components as well as overarching values, health policy, and online technologies need to be adapted to reduce the death count and meet the evolving needs of marginalised individuals who use opioid. Future research should focus on scientifically assessing such implementations to guide evidence-based decision making.

## Introduction

 The COVID-19 pandemic necessitated fast responses and adaptations from hospitals and healthcare systems worldwide. This is exemplified in the article by Hodgins et al^1^ who developed the COVID-19 System Shock Framework (CSSF), building on previous theoretical work by Hanefeld and colleagues.^2^ The framework comprises of five core dimensions, “health services,” “health workforce,” “information systems,” “products and technologies,” and “funding and finance,” that intersect with two additional overarching factors: “health system values” and “health policy and governance” (see Table). Core dimensions are to be understood as the essential parts of a functioning healthcare system, whereas the overarching factors shape the changes made to the core dimensions during a system shock and affect how these changes are experienced and perceived on an individual and a community level.

**Table:** The COVID-19 System Shock Framework by Hodgins et al^1^

	**↓ System Shock ↓**	
Health system values	Health services	Health policy and governance
	Health workforce	
	Information systems	
	Products and technologies	
	Funding and finance	
	Resilient health system	

 Hodgins et al apply the CSSF in order to assess the innovations and changes created in response to the pandemic by the Sydney Children’s Hospitals Network (SCHN), the largest provider of children’s health services in the Southern hemisphere. Their article demonstrates the analytical value of their framework and illustrates the responses implemented by SCHN to address the COVID-19 pandemic. Perhaps most importantly, their system’s resilience to COVID-19 was determined by proactivity rather than reactivity, by their ability to innovate, as outlined by the CSSF, rather than merely coping with the COVID-19 pandemic.

 Given the resilience of the SCHN to the COVID-19 system shock, it provides valuable takeaways for other health systems in different contexts. North America has been experiencing its own system shock in the past years and has so far not been able to adequately address the overdose crisis. Indeed, overdose deaths in the United States are steadily increasing, with a death count that exceeded 100 000 in 2021.^3^ However, this steady increase also marks a key difference to the more sudden COVID-19 system shock. Whereas the latter was responded to in a short timeframe, the “fentanyl system shock” occurred in a resilient system of treatment and harm reduction services for substance use disorders.^4^

## Applying the CSSF Factors to the Fentanyl System Shock

*Health services:* expanding access to and retention in existing treatment options, creating more appropriate treatment options and developing harm reduction services. The increasing significance of fentanyl does not only require reshaping and expanding existing health services but also requires new innovative developments and novel treatment approaches.^5,6^ This includes the adaptation of health services to evolving substance use patterns. Given today’s opioid use patterns being dominated by fentanyl, evaluating a novel treatment option called fentanyl-assisted treatment, which is analogous to heroin-assisted treatment, has been suggested in order to adequately address the needs of individuals with illicit fentanyl use and reduce barriers to treatment.^7^ This alone is insufficient though, and expanding access to evidence-based treatment options and low-barrier harm reduction services (eg, supervised consumption rooms) is critical.

*Health workforce:* developing an interdisciplinary system response and diversifying the staff involved in the care of those who use fentanyl. The combined efforts of healthcare personnel comprising of nurses, social workers, psychologists, and physicians improves health outcomes and allows for the integrated treatment of dual diagnoses, trauma, and social harms.^8^ Treating the underlying substance use disorder in isolation is not enough to account for patients’ various needs; physical, mental, and social stabilisation is made possible with individualised comprehensive care that incorporates all aspects of an individual’s life, and the healthcare force must reflect this.

*Information systems: *developing publicly available websites and dashboards about number of non-fatal and fatal overdose deaths, treatment statistics (eg, retention, drop-out) as well as recent illicit drug supply mixtures, integrated with accessible drug checking services. Existing data on trends and developments of fentanyl use should be public information and easily available. Information systems would allow drug checking services, which assess the purity and contamination of illicit substances, to publish their results and be kept updated as drug markets evolve. Additionally, current data on non-fatal and fatal overdoses, treatment intake, treatment retention, and treatment drop-out, among other statistics, should be made available on interactive digital dashboards. For instance, the total number of COVID-19 infections, intensive care unit admissions, vaccinations, and deaths were all available to the public. A similar approach for the overdose crisis may be warranted.

*Medical products and technology:* implementing online technologies, evaluating novel products like medication vending machines and higher doses of naloxone to address high-potency opioids. eMentalHealth solutions could allow patients to more easily access treatment and harm reduction services, while also being in better contact with their healthcare team (eg, in case of an emergency or for prescription adjustments). For instance, the Risk Assessment and Management Platform is an initiative currently developed in Vancouver, Canada as a comprehensive platform addressing the needs of high-risk users.^9^ However, websites and smartphone apps have so far not reached their full potential; more funding and effectively implementing these solutions is needed. Furthermore, privacy concerns and data protection laws may prove to become major obstacles when attempting to implement such platforms. Economic challenges like homelessness may also prevent these systems to reach their target population. Moreover, the introduction of mySafe vending machines for hydromorphone dispensing in Canada is an interesting technological response to the overdose crisis, but its impact remains to be evaluated.^10^

*Funding and finance:* investing in economically worthwhile responses which will reduces cost to the overall healthcare system, as well as to other systems (eg, judicial system). The long-term economic benefit of a successful response to the overdose crisis should be considered during the creation and budgeting of overdose funds. Emergency room visits following an overdose, treatment of chronic infections and health complications among individuals, and criminal justice system involvement leads to significant public expenditure.^11^ Much of this cost can be avoided with the implementation of effective long-term treatment approaches such as effective opioid agonist treatment. Although this may be expensive to build from the ground up, they have shown to be cost-effective in the long-term due to increased adherence and beneficial health and social outcomes.^12^

*Health system values:* listening to the needs of patients and advocating for care that directly aligns with their goals and preferences. Moral judgements, prejudice and a “father know best attitude” must be replaced by flexible shared decision making between patients and their healthcare team, that allow for the pursuit of patient-centred goals (eg, professional, personal, and social). For instance, enabling equity of access to care and enabling patient’s autonomy can be facilitated with remote care or take-home medication on a case-by-case basis. As it stands, some treatment options (eg, heroin-assisted treatment; injectable opioid agonist treatment) require patients to visit treatment centres daily or twice daily for supervised injections, which can discourage individuals from seeking treatment.^13^ Although these regulations were somewhat relaxed recently, a more fundamental reform of these policies might be warranted.^14^

*Health policy and governance: *adapting policies to substance use trends and needs of individuals, developing policies using actual evidence, and communicating successful and non-successful efforts transparently to the public. Expertise drawn from experts from various disciplines, patients, and communities (eg, families) should inform policy and guide governance, structured around evidence-based treatment options and harm reduction services using long-term large-sample gold-standard research designs.

## Online Technology – the Most Important and Interconnecting Factor?

 Along with the five core factors and two overarching factors taken from the CSSF presented by Hodgins et al, another important and interconnecting factor prompted us to adapt the model for our conceptual Fentanyl System Shock Framework: online technology. Efforts in British Columbia have so far been unsuccessful at reducing mortality and hospitalisation rates due to overdose.^15^ Though an assortment of digital options and online technologies are already somewhat available, there is still a large gap to fill. Health services need to be accessible digitally and merge with healthcare provision itself. Scheduling an appointment with the treating physician for common requests like minor dose adjustments could become obsolete if a digital communication solution is established. Moreover, smart devices (eg, watches) could help monitor and document vital functions. This could then be made immediately available to the healthcare staff (with the patient’s consent) and allow alerts to be sent to the healthcare providers if there are any sudden changes. Apps could allow to set a digital medication intake schedule with reminders if the patient wishes to receive them. Any adjustments to the patient’s treatment regimen could automatically synchronise to the patients’ devices and update their digital medication intake schedule. Withdrawal and overdose symptoms could be intuitively documented by the user through personalised interfaces, providing comprehensive charts and statistics over time. Digitalisation with a strong focus on user consent and data security would put the treatment regimen quite literally in the patient’s pocket, therefore maximising patient autonomy.

 Online technologies are also the fastest and most convenient way to spread information, which is particularly important in rapidly changing environments like drug markets with emerging street drugs. As previously laid out, digital dashboards can combine public and professional discourse with the presentation of live statistics, which can have a tremendous impact on motivation, work ethic, performance, and research. Equally, making drug checking results publicly available would empower individuals who use drugs. Pictures of products and their packaging, information about specific mixes found on the drug market, and estimates of potency could help with risk assessment and management.

 Finally, there must be a willingness to replace stale inefficient systems and organisational structures with up-to-date efficient digital solutions. Many services and measures that are already being provided like drug checking and data collection do not require radical changes, but rather digital enhancement. Availability of data massively increases the value of already existing services. However, merging them with online solutions by implementing novel technologies requires an open mindset and the awareness that the system as it currently stands has proven incapable of addressing the overdose crisis.

## Conclusion

 Hodgins et al described the CSSF, a clear and structured analysis of the SCHN response to the challenges of the COVID-19 pandemic.^1^ From the CSSF, we identified major take-aways for healthcare systems in North America in addressing the overdose crisis. In particular, expanding and innovating health services, diversifying the workforce, maximising information systems, evaluating and implementing new products and technologies, funding long-term solutions, prioritising patient’s needs and preferences, and adapting policy and governance accordingly and transparently, would all allow for increased resilience to the system shock created by fentanyl, as well as to high-potency opioids moving forward. We also add to this conceptual model with online technologies as a third overarching factor, which as untapped potential and immense capacity. Online technologies can allow for the collection of data from patients and the healthcare system, sharing of data with patients, their communities, the health workforce, or the public, and can be adapted quickly according to the changing dynamics of the overdose crisis. Developing a framework in response to the fentanyl system shock might prove useful in achieving successful implementations of countermeasures to the current overdose crisis. Such a development would require an openness for change and the provision of funding on a system level. Indeed, since 2017 the Canadian government provided $800 million in funding to tackle the current crisis, underlining the political will to financially support strategies to increase the system’s resilience.

 Notably, the dialogue of this commentary is purely theoretical at this point in time. Any actual system changes would have to be subject of thorough scientific evaluation, as even the most well-intended measures might come with unforeseen consequences and could even decrease the resilience of the respective health system.

 North America is in desperate need of a paradigm shift to respond to the fentanyl system shock, as current measures have been and currently are unsuccessful to reduce the rising mortality rate. The CSSF model developed by Hodgins et al can be projected and adapted to the overdose crisis and fentanyl system shock, and used to analyse the innovative changes required in health systems and determine the coordination and integration at various levels of the system to create and sustain changes.

## Ethical issues

 Not applicable.

## Competing interests

 Authors declare that they have no competing interests.

## Authors’ contributions

 MM, JNW, and RMK conceptualised and drafted the manuscript.
